# Effects of Transglutaminase on the Texture and Consumer Acceptability of Reduced‐Fat and Reduced‐Sodium Pork Balls

**DOI:** 10.1155/ijfo/8105626

**Published:** 2026-11-04

**Authors:** Jarinya Sutti, Sujinda Sriwattana, Napapan Chokumnoyporn, Witoon Prinyawiwatkul

**Affiliations:** ^1^ Product Development Technology Division Faculty of Agro-Industry Chiang Mai University Chiang Mai Thailand cmu.ac.th; ^2^ Culinary Art and Kitchen Management Division Faculty of Science Chandrakasem Rajabhat University Bangkok Thailand; ^3^ School of Nutrition and Food Sciences Louisiana State University Agricultural Center Baton Rouge Louisiana USA lsu.edu

**Keywords:** consumer acceptability, health foods, pork balls, reduced-fat meat products, reduced-sodium meat products, transglutaminase

## Abstract

Transglutaminase (TGase) is widely used to improve the texture and functional properties of processed meat products. However, information on the appropriate TGase concentration for maintaining desirable quality in reduced‐fat and reduced‐sodium pork products remains limited. This study investigated the effects of TGase incorporation at four levels (0%, 0.1%, 0.2%, and 0.3% w/w of pork leg meat) on the physicochemical properties, textural characteristics, and consumer acceptability of reduced‐fat and reduced‐sodium pork balls using a randomized complete block design. Increasing the TGase concentration generally enhanced the textural properties of the pork balls. The formulation containing 0.1% TGase received the highest texture liking score (6.8) and overall liking score (6.5). Its hardness, springiness, cohesiveness, and chewiness were 12,273.19 g force, 0.68, 0.52, and 6,370.55 N mm, respectively. Based on its favorable sensory performance, the 0.1% TGase formulation was selected for further validation. The validated product was microbiologically safe, with consumer acceptability scores ranging from 6.5 to 6.9. Furthermore, the product contained 6.46 g fat and 337.7 mg sodium per 100 g, representing reductions of 47.5% in fat and 27.1% in sodium compared with the reference product. These reductions met the United States Food and Drug Administration criteria for “reduced‐fat” and “reduced‐sodium” nutrient content claims. Overall, incorporation of 0.1% TGase effectively improved the textural and sensory quality of reduced‐fat and reduced‐sodium pork balls and shows potential for the development of healthier processed meat products.

## 1. Introduction

Processed meat products are widely consumed because of their convenience, desirable sensory characteristics, and nutritional value. However, many processed meat products contain relatively high levels of sodium and fat, which are commonly added to achieve desirable flavor, texture, juiciness, water‐holding capacity, and processing stability [1, 2]. Sodium chloride (NaCl) contributes to saltiness and flavor, promotes extraction and solubilization of myofibrillar proteins, and consequently contributes to the formation of a stable meat protein matrix [3]. Fat contributes to flavor, juiciness, tenderness, and texture and can improve water‐holding capacity and reduce cooking loss in processed meat products [4]. Despite these technological functions, excessive dietary sodium and fat intake has been associated with an increased risk of diet‐related health problems, including hypertension, cardiovascular disease, and obesity [5–10]. Therefore, the development of processed meat products with reduced sodium and fat while maintaining desirable quality characteristics has become an important research and product‐development objective.

Various approaches have been investigated to reduce sodium and fat in processed meat products. Sodium reduction strategies include partial replacement of NaCl with other mineral salts, incorporation of flavor enhancers, modification of salt distribution, and the use of alternative processing technologies [6, 11–18]. Fat reduction can be achieved by partially or completely replacing animal fat with fat replacers, fat substitutes, fat mimetics, or other functional ingredients capable of maintaining desirable physicochemical and sensory properties [9, 19–23]. However, simultaneous reduction of both sodium and fat is technically challenging because these ingredients perform multiple functions in meat systems. Reducing both components may result in weaker protein networks, altered water retention, reduced juiciness, and undesirable textural characteristics. Thus, technological approaches that can compensate for these quality losses are needed.

Pork balls are popular emulsion‐type processed meat products in many Asian countries, including Thailand, China, Japan, and Vietnam [24–30]. Conventional pork balls are commonly prepared from pork leg meat combined with pork back fat, together with salt, water or ice, and other functional ingredients. Pork leg meat is an important lean‐meat component because it provides myofibrillar proteins that contribute to the formation of the heat‐induced protein gel network responsible for the structure and texture of pork balls. In contrast, pork back fat provides lipid functionality, contributing to flavor, juiciness, lubrication, and desirable mouthfeel. The incorporation of pork back fat also influences the emulsion stability and textural characteristics of the final product. Therefore, reducing the proportion of pork back fat while maintaining an acceptable meat protein network is a particular technological challenge in the development of reduced‐fat pork balls.

Transglutaminase (TGase; protein‐glutamine *γ*‐glutamyltransferase, EC 2.3.2.13) is an enzyme that catalyzes the formation of covalent *ε*‐(*γ*‐glutamyl) lysine bonds between proteins [31]. In meat systems, TGase can promote protein cross‐linking and strengthen the three‐dimensional protein network, thereby improving gel strength, binding properties, and textural characteristics. The application of TGase has therefore been investigated as a means of improving the texture of various processed meat products and potentially reducing the dependence on conventional functional ingredients such as salt and phosphate [32–34]. However, excessive TGase incorporation may produce an excessively firm texture and does not necessarily result in improved consumer acceptance. Consequently, determining an appropriate TGase concentration is important for achieving a balance between technological functionality and sensory quality.

Although previous studies have demonstrated the textural benefits of TGase in different meat products, limited information is available regarding the application of TGase specifically to pork balls formulated with simultaneous reductions in both fat and sodium. In particular, the relationship between TGase concentration, instrumental texture, protein characteristics, and consumer acceptability in such a formulation has not been fully established.

The innovativeness of this study lies in the integrated use of TGase to overcome the quality limitations associated with the simultaneous reduction of fat and sodium in pork balls. Rather than evaluating TGase solely as a texture‐enhancing enzyme, this study investigated its potential to maintain desirable product characteristics within a healthier formulation containing reduced levels of two major dietary components of concern. Furthermore, the study linked enzyme concentration with instrumental texture, consumer acceptability, protein characteristics, microbial safety, and nutritional and regulatory validation. This integrated approach provides practical information for the development of acceptable reduced‐fat and reduced‐sodium pork balls and demonstrates the potential application of TGase as a technological tool for producing healthier processed meat products.

## 2. Material and Method

### 2.1. Materials and Preparation for Reduced‐Fat and Reduced‐Sodium Pork Balls

Reduced‐fat and reduced‐sodium pork ball products were produced based on the formulations from our previous research [35] as illustrated in Table 1. Fresh pork leg meat and pork back fat (Charoen Pokphand Foods Public Co. Ltd, Thailand) were separately minced through a 5‐mm plate (Electrolux, China). Minced pork leg was mixed with sodium tripolyphosphate (Aditya Birla Chemicals (Thailand) Co. Ltd., Thailand). These minced meat and back fat were covered and stored at 4°C for 24 h. The minced meat was then mixed with the other ingredients shown in Table 1 in a bowl chopper (RS 20, Josef Meissner GmbH. and CO. KG., Koln, Germany), followed by addition of minced back fat. During mixing, crushed ice was added to keep the temperature below 18°C. All ingredients were chopped for 9 min. The meat batter was shaped into a small ball then put in warm water at 60°C for 10 min, then in water at 80°C for 15 min before passing it through cold water for 10 min. The pork balls were vacuum packed in bags and stored at 4°C until use.

**Table 1:** Formulations ^∗^ of reduced‐fat and reduced‐sodium pork balls containing various concentrations of transglutaminase enzyme.

Ingredients %	Treatment 1 (T1)	Treatment 2 (T2)	Treatment 3 (T3)	Treatment 4 (T4)
Fresh pork leg meat	100.00	100.00	100.00	100.00
Pork back fat^*1^	9.40	9.40	9.40	9.40
Inulin^*2^	13.00	13.00	13.00	13.00
NaCl^*3^	2.50	2.50	2.50	2.50
KCl^*4^	1.70	1.70	1.70	1.70
Pepper^*^	0.30	0.30	0.30	0.30
Garlic^*^	3.30	3.30	3.30	3.30
STPP^*^	0.10	0.10	0.10	0.10
Ice^*^	33.00	33.00	33.00	33.00
TGase^*^	0.00	0.10	0.20	0.30

*Note:*  ^∗^Weight percentage of fresh pork leg meat. ^1, 2, 3, 4^ A range (%) of each ingredient (7.00–10.97; 11.58–13.00; 2.17–2.60; 1.55–2.68, respectively) used to make pork balls [35].

Abbreviations: Activa TG‐AK, transglutaminase activity 50–84 U/g; STPP, sodium tripolyphosphate; TGase, transglutaminase enzyme.

The TGase enzyme was purchased from Ajinomoto (Thailand) Co. Ltd., Bangkok, Thailand, sodium tripolyphosphate from Aditya Birla Chemicals (Thailand) Co. Ltd., Bangkok, Thailand, NaCl from Thai Refined Salt Co. Ltd., Bangkok, Thailand, and KCl from Stan Chem international limited, Peterborough, England. Other ingredients were purchased from local grocery stores.

### 2.2. Experimental Design

The completely randomized design (CRD) was used in this research. Four levels of TGase (0%, 0.1%, 0.2%, and 0.3% by weight of the fresh pork leg meat, designated as T1, T2, T3, and T4, respectively) were added with other ingredients (Table 1) during mixing process. Treatment 1 (T1; no TGase) was used as the control.

### 2.3. Physiochemical Analysis

Before analysis, the stored pork ball samples were heated in boiling water for 3 min and subsequently maintained in an incubator at 70°C until texture analysis.

The textural profile analysis (TPA) of the reduced‐fat and reduced‐sodium pork balls was performed using a TA‐XT Plus texture analyzer (Texture Technologies Corp., Scarsdale, New York, United States) equipped with a 50‐mm‐diameter aluminum cylindrical probe, following the method described by Bourne [36]. Each pork ball was subjected to a double‐compression cycle to 75% of its original height to simulate the first and second bites during mastication. The pretest and posttest speeds were 1.0 and 3.0 mm/s, respectively. The TPA parameters were calculated as follows: hardness (*H* *d*), the maximum force required during the first compression (*N*); cohesiveness (*C* *h*), the ratio of the area under the second compression curve to that under the first compression curve (dimensionless); springiness (*S* *p*), the extent to which the sample recovered its original height after the first compression; and chewiness (*C* *w*), calculated as *H* *d* × *C* *h* × *S* *p* (*N* · *m* *m*). Ten replicate measurements were performed for each treatment.

The pH of all samples was analyzed in duplicate using a pH meter (Thermo Orion 520A, Waltham, Massachusetts, United States). Analysis of protein subunits was performed in duplicate using SDS‐PAGE. Five grams of pork ball sample was solubilized with 20 mL of the respective electrophoresis extraction buffer and incubated at 37°C for 1 h. The sample was then centrifuged to separate the insoluble fraction, and the resulting clear supernatant was collected. The supernatant was dialyzed against distilled water for 12 h, with the distilled water replaced every 2 h. Following dialysis, the sample was centrifuged at 10,000 rpm for 3 min, and the resulting supernatant was collected and concentrated approximately tenfold using a vacuum evaporator. The total protein content of the extracts was determined using the Lowry assay [37], and the protein extracts were adjusted to the same protein concentration prior to electrophoresis.

For SDS‐PAGE, the protein extracts were mixed with a loading dye [10% (w/v) sodium dodecyl sulfate (SDS), 0.02% (w/v) bromophenol blue, and 10% (v/v) glycerol] at a ratio of sample:loading dye (2:1), and heated to 80°C for 4 min. No reducing agent, such as *β*‐mercaptoethanol or dithiothreitol (DTT), was added to the loading dye; therefore, electrophoresis was performed under nonreducing conditions. Electrophoresis was performed using a 4.5% stacking gel and a 7.5% resolving gel in gel electrophoresis systems (BIO‐RAD, Hercules, California, United States) [38]. Equal volumes (15 *μ*L) of each protein sample together with a prestained protein molecular weight marker were loaded into each well. Electrophoresis was carried out in Tris–glycine–SDS running buffer at 100 V for approximately 2 h until the tracking dye reached the bottom of the gel. Then gel was stained with the mixture of Comassie blue for 60 min at 37°C, washed with Solution I for 30 min at 37°C and in Solution II for 30 min at 37°C [39]. The gel sheet was destained until the blue bands appeared clearly. Gel images were taken using a digital camera over a light box. A protein molecular weight marker (53–220 kDa; GE Healthcare United Kingdom Ltd., United Kingdom) was used as the molecular mass standard.

### 2.4. Consumer Test

The sensory acceptability of the pork ball samples was evaluated under controlled conditions at the Sensory Evaluation and Consumer Testing Unit, Faculty of Agro‐Industry, Chiang Mai University (CMU), Thailand, using the SuSense computer‐based sensory evaluation program (Thailand). A total of 100 consumers were recruited from the CMU campus. Participants were 18–60 years old, regularly consumed pork and pork products, and reported no known allergies to pork or any ingredients used in the pork ball formulations. Before participating in the evaluation, all consumers provided informed consent and were informed of the potential risks associated with consumption of the test products.

Each consumer evaluated four pork ball formulations and rated overall liking, salty taste, overall taste, and texture using a 9‐point hedonic scale (1 = *dislike extremely*, 5 = *neither like nor dislike*, and 9 = *like extremely*) [40]. For sample preparation, pork balls were boiled in water for 3 min and placed in individual plastic cups labeled with randomly assigned three‐digit codes. The samples were maintained at 70°C under controlled conditions until serving. Sample presentation order was balanced and randomized among consumers to minimize potential order and carry‐over effects. Drinking water was provided for palate cleansing between sample evaluations. Each participant received a small token of appreciation for participation in the study.

### 2.5. Validation Study of the Selected Reduced‐Fat and Reduced‐Sodium Pork Ball Containing 0.1% TGase

The pork ball formulation containing 0.1% TGase, which received the highest consumer acceptability in the preliminary consumer test described in Section 2.4, was selected for further validation. The selected formulation was evaluated for physicochemical properties, proximate composition, sodium content, microbial safety, and consumer acceptability.

The pH was determined in duplicate, and TPA was performed in 10 replicates using the procedures described in Section 2.3. Proximate composition, including moisture, protein, ash, and lipid contents, was determined in duplicate according to the methods of the Association of Official Analytical Chemists (AOAC) [41]. Carbohydrate content was calculated by difference, where applicable.

Sodium content was determined in triplicate according to the methods [42–44] with slight modifications. Briefly, 1 g of dried pork ball sample was weighed into a lidded Erlenmeyer flask, and 20 mL of a nitric acid/sulfuric acid mixture (6:1, v/v) was added. The sample was mixed and allowed to stand at room temperature for 24 h for predigestion. The sample was then heated on a hot plate at 150°C until digestion was complete and the solution had evaporated to dryness. The resulting digest was transferred quantitatively into a 10‐mL volumetric flask and dissolved using hydrochloric acid/deionized water (1:4, v/v). Sodium concentration was determined using an atomic absorption spectrometer (Avanta M1, Hampshire, Illinois, United States). Quantification was based on a calibration curve prepared using sodium standard solutions at concentrations ranging from 0.40 to 1.50 ppm. The sodium content was expressed as mg/100 g of pork ball.

Microbiological quality and safety were evaluated according to the methods described in the United States Food and Drug Administration Bacteriological Analytical Manual (FDA BAM) [45]. The analyses included total plate count and detection/enumeration of *Salmonella* spp., *Escherichia coli*, *Staphylococcus aureus*, and *Clostridium perfringens*.

A total of 200 consumers were recruited for the consumer test validation. Participants evaluated the developed pork ball for overall liking, salty taste, overall taste, and texture using the same 9‐point hedonic scale and evaluation procedures described in Section 2.4. The validation test was conducted to confirm the consumer acceptability of the selected 0.1% TGase formulation under the same controlled sensory evaluation conditions.

### 2.6. Statistical Analysis

The experiments were conducted with the numbers of replicates specified for each analysis, and the results are expressed as mean ± standard deviation. Data on physicochemical properties and textural characteristics were analyzed by one‐way analysis of variance (ANOVA) using a CRD. Consumer acceptability data were analyzed by ANOVA using a randomized complete block design (RCBD), with consumers treated as blocks. When significant differences among treatment means were detected, mean comparisons were performed using the least significant difference (LSD) test. Statistical significance was accepted at *p* < 0.05. All statistical analyses were performed using SPSS Version 11.0 (SPSS Inc., Chicago, Illinois, United States).

## 3. Results and Discussion

### 3.1. Effects of TGase on Qualities of Reduced‐Fat and Reduced‐Salt Pork Balls

Results from our previous study [35] demonstrated that reduced‐fat and reduced‐sodium pork balls had inferior textural properties and sensory acceptability compared with commercial pork ball products available in the Thai market. This may be attributed to the important technological functions of fat and salt in processed meat products, including their contributions to texture, water‐holding capacity, gel formation, and overall palatability [46, 47]. Consequently, simultaneous reduction of fat and sodium can compromise the structural and sensory quality of pork balls. Therefore, in the present study, TGase was incorporated as a functional enzyme to strengthen the protein network and improve the textural quality and consumer acceptability of the reduced‐fat and reduced‐sodium pork ball formulation.

Texture profile analysis (TPA) results (Table 2) showed that increasing the TGase concentration generally improved the textural properties of the reduced‐fat and reduced‐sodium pork balls, as reflected by increases in hardness, springiness, cohesiveness, and chewiness. This improvement can be attributed to the catalytic activity of TGase, which promotes the formation of covalent *ε*‐(*γ*‐glutamyl) lysine cross‐links between meat proteins, thereby facilitating the development of a stronger and more cohesive protein network [48–50]. The formation of this cross‐linked protein matrix may help compensate for the reduced structural and water‐binding functionality associated with lowering fat and NaCl levels in conventional pork ball formulations. The observed improvement in texture (Table 2) may be partially associated with changes in the protein profile revealed by SDS‐PAGE (Figure 1), where protein band intensity increased with increasing TGase concentration.

**Table 2:** Instrumental textural parameters  ^∗^ of reduced‐fat and reduced‐sodium pork balls containing various concentrations of transglutaminase enzyme.

Treatments	Hardness (*g*force)	Springiness	Cohesiveness	Chewiness (*N*.mm)
T1 (TGase 0.0%)	10,786 ± 1360^c^	0.68 ± 0.05^b^	0.48 ± 0.02^b^	5229 ± 761^c^
T2 (TGase 0.1%)	12,273 ± 1756^b^	0.69 ± 0.03^b^	0.52 ± 0.02^a^	6371 ± 1003^b^
T3 (TGase 0.2%)	14,038 ± 2237^a^	0.75 ± 0.03^a^	0.53 ± 0.02^a^	7461 ± 1247^a^
T4 (TGase 0.3%)	15,081 ± 1847^a^	0.77 ± 0.03^a^	0.52 ± 0.02^a^	7842 ± 1164^a^

*Note:*  ^∗^Mean ± standard deviation based on 10 replications. Means within each column followed by different letters are significantly different (*p* < 0.05).

Abbreviations: Activa TG‐AK, transglutaminase activity 50–84 U/g; TGase, transglutaminase enzyme.

**Figure 1:**
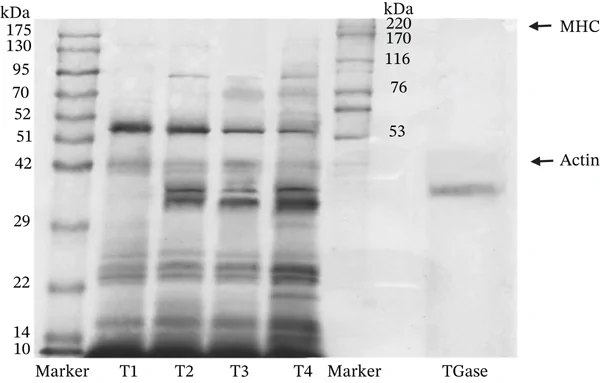
SDS‐PAGE of each reduced‐fat and reduced‐sodium pork ball formulations containing various concentrations of transglutaminase enzyme, TGase; T1 (0% TGase), T2 (0.1% TGase), T3 (0.2% TGase), and T4 (0.3% TGase).

Among the treatments, samples containing 0.1% TGase showed significantly higher hardness and cohesiveness values than the control without TGase, indicating that even a low level of enzyme incorporation was effective in improving product texture. Similar findings were reported by Tseng [48], who observed increased gel strength in chicken balls with increasing TGase concentrations. In the present study, however, no significant differences (*p* ≥ 0.05) in most texture parameters were observed between samples containing 0.2% and 0.3% TGase (Table 2). This finding may suggest that the available reactive protein sites became saturated at higher enzyme concentrations, limiting further cross‐linking reactions and texture improvement.

The increased chewiness and springiness observed in TGase‐treated samples are desirable characteristics for emulsion‐type meat balls, particularly in Asian markets where elastic and firm texture is preferred by consumers. Nevertheless, excessive increases in hardness may negatively affect sensory acceptability. Therefore, optimization of TGase concentration is important to achieve a balance between improved texture and consumer preference. Overall, the results indicate that TGase can effectively improve the texture characteristics of reduced‐fat and reduced‐sodium pork balls and may serve as a promising technological approach for developing healthier processed meat products without compromising product quality.

The total protein content of the reduced‐fat and reduced‐sodium pork balls ranged from 6.11 to 6.92 mg/mL (Table 3). No significant differences (*p* ≥ 0.05) were observed among the samples containing 0.0%–0.2% TGase. However, the sample containing 0.3% TGase showed a significantly lower measured protein content than the other treatments. This reduction may be related to the protein cross‐linking promoted by TGase at the highest concentration. The formation of covalent protein aggregates may alter protein solubility or the accessibility of reactive groups involved in the Lowry reaction, thereby resulting in a lower apparent protein concentration. Therefore, the lower value observed at 0.3% TGase should be interpreted as a difference in measurable protein rather than a reduction in the actual protein content of the product.

**Table 3:** Total protein content and pH of reduced‐fat and reduced‐sodium pork balls containing various concentrations of transglutaminase enzyme.

Treatments	Total protein (mg/ml) ^∗^	pH values ^∗^
T1 (TGase 0.0%)	6.695 ± 0.16^a^	5.87 ± 0.08^b^
T2 (TGase 0.1%)	6.918 ± 0.10^a^	6.13 ± 0.02^a^
T3 (TGase 0.2%)	6.780 ± 0.21^a^	6.13 ± 0.01^a^
T4 (TGase 0.3%)	6.112 ± 0.11^b^	6.14 ± 0.00^a^

*Note:*  ^∗^Mean ± standard deviation based on duplications. Means within each column followed by different letters are significantly different (*p* < 0.05).

Abbreviations: Activa TG‐AK, transglutaminase activity 50–84 U/g; TGase, transglutaminase enzyme.

The pH values of pork balls containing TGase were significantly higher than those of the control sample without TGase (6.13–6.14 vs. 5.87; Table 3). In general, postmortem pork muscle exhibits a pH lower than that of living muscle due to glycolysis and lactic acid formation, while the preferred pH range for processed pork products is approximately 6.3–6.7 [51]. The increased pH observed in TGase‐treated samples may be attributed to the alkaline nature of the TGase preparation, which reportedly possesses a pH range of approximately 11.0–13.0 [52, 53]. Similar observations have been reported in low‐fat chicken meat gels incorporated with microbial TGase, where enzyme‐treated samples showed slightly higher pH values than control samples without TGase [52].

The increase in pH may also contribute positively to the functional properties of the pork balls, particularly water‐holding capacity and protein gel formation, thereby supporting the improved texture characteristics observed in TGase‐treated samples. Nevertheless, the relatively similar pH values among TGase‐treated formulations suggest that increasing TGase concentration above 0.1% had minimal additional effect on product acidity or alkalinity.

The SDS‐PAGE analysis results of pork balls are given in Figure 1. SDS‐PAGE was used as a separation technique to differentiate and dissociate meat proteins based on their size [54]. In the present study, the addition of microbial TGase altered the SDS–PAGE protein‐banding pattern. Specifically, the intensity of protein bands in the molecular weight range of approximately 51–52 kDa gradually decreased with increasing TGase concentration, whereas protein bands in the 29–42 kDa range became more prominent. These findings are consistent with the catalytic mechanism of TGase, which promotes the formation of *ε*‐(*γ*‐glutamyl)lysine covalent cross‐links between the *γ*‐carboxamide group of glutamine and the *ε*‐amino group of lysine residues. This enzymatic cross‐linking modifies the molecular organization of myofibrillar proteins and consequently alters their electrophoretic mobility during SDS–PAGE [55–58]. However, no protein accumulation was observed at the gel loading well or in the region above 250 kDa, which is commonly regarded as evidence of myosin heavy chain (MHC) polymerization in TGase‐treated meat systems. Therefore, although the observed changes in protein band intensity were consistent with TGase‐mediated cross‐linking, the present results were insufficient to directly confirm extensive MHC polymerization. Two protein bands within the molecular weight range of 29–42 kDa were consistently detected in all TGase‐treated samples, and their intensities increased with increasing enzyme concentration. Although Aktaş and Kılıç [57] reported that microbial TGase primarily promotes the formation of high‐molecular‐weight protein polymers while reducing the intensity of proteins with intermediate molecular weights through *ε*‐(*γ*‐glutamyl)lysine cross‐linking, the increased intensity observed in the present study may reflect structural rearrangements of the protein network. Such rearrangements could enhance the separation or detection of specific proteins or protein subunits within the 29–42 kDa region during SDS–PAGE. Nevertheless, the identities of these protein bands cannot be confirmed solely by electrophoretic mobility and require further characterization using techniques such as Western blotting or LC–MS/MS. The gradual reduction in the intensity of protein bands at 51–52 kDa, accompanied by the appearance of faint bands at slightly higher molecular weights, may indicate progressive protein cross‐linking and the formation of larger protein complexes. TGase catalyzes the formation of *ε*‐(*γ*‐glutamyl)lysine covalent bonds between glutamine and lysine residues, thereby promoting the conversion of protein monomers into higher molecular weight polymers. As a consequence, fewer monomeric proteins are able to migrate into the resolving gel, resulting in decreased band intensity in the 51–52 kDa region. These observations are consistent with the findings of Aktaş and Kılıç [57], who reported that the addition of microbial TGase to minced meat induced cross‐linking among myofibrillar proteins, leading to a reduction in protein bands of intermediate molecular weight and the accumulation of high‐molecular‐weight protein polymers at the gel loading well or their inability to migrate into the gel. Similarly, Tseng et al. [58] demonstrated that TGase treatment reduced the intensity of medium‐molecular‐weight protein bands while promoting the formation of high‐molecular‐weight protein aggregates that remained at the top of the gel.

The results of the consumer acceptability test (Table 4) showed that TGase concentration significantly affected the sensory characteristics of the reduced‐fat and reduced‐sodium pork balls. All samples received mean acceptability scores above 5.0 for the evaluated attributes, indicating that the products were generally acceptable to consumers across the tested TGase concentrations. However, the addition of TGase at 0.1%–0.2% generally resulted in higher sensory scores, whereas increasing the concentration to 0.3% tended to decrease consumer acceptability.

**Table 4:** Sensory acceptability scores ^∗^ of reduced‐fat and reduced‐sodium pork balls containing various concentrations of transglutaminase enzyme.

Treatments	Overall liking	Salty	Overall taste	Texture
T1 (TGase 0.0%)	5.7 ± 1.3^b^	5.2 ± 1.6^b^	5.6 ± 1.4^b^	5.7 ± 1.4^b^
T2 (TGase 0.1%)	6.5 ± 1.4^a^	6.3 ± 1.5^a^	6.4 ± 1.4^a^	6.8 ± 1.3^a^
T3 (TGase 0.2%)	6.2 ± 1.3^ab^	6.1 ± 1.3^a^	6.2 ± 1.2^a^	6.2 ± 1.4^b^
T4 (TGase 0.3%)	5.8 ± 1.6^b^	5.4 ± 1.9^b^	5.6 ± 1.7^b^	6.0 ± 1.6^b^

*Note:*  ^∗^Mean ± standard deviation based on 100 consumer responses and on a 9‐point hedonic scale (1 = *dislike extremely*, 5 = *neither dislike nor like*, and 9 = *like extremely*; [40]). Means within each column followed by different letters are significantly different (*p* < 0.05).

Abbreviations: Activa TG‐AK, transglutaminase activity 50–84 U/g; TGase, transglutaminase enzyme.

The pork balls containing 0.1% TGase received the highest texture liking score (6.8) among all treatments. The scores for overall taste (6.4), salty taste (6.3), and overall liking (6.5) were not significantly different from those of the 0.2% TGase treatment but were significantly higher than those of the control and 0.3% TGase treatments. The higher texture acceptability of the 0.1% TGase treatment may be associated with the formation of a more cohesive protein network through TGase‐mediated cross‐linking, which improved the textural characteristics of the pork balls. However, the further increase in TGase concentration to 0.2%–0.3% resulted in progressively higher instrumental hardness and chewiness, whereas sensory acceptance did not increase accordingly. In particular, the lower acceptability observed at 0.3% TGase may indicate that excessive protein cross‐linking produced a texture that was less desirable to consumers. These findings highlight the importance of balancing the extent of TGase‐induced protein cross‐linking with sensory preferences when developing reduced‐fat and reduced‐sodium pork balls.

Although texture parameters from instrumental analysis continued to increase with higher TGase concentrations (Table 2), sensory acceptability did not improve correspondingly (Table 4). Samples containing 0.3% TGase exhibited lower overall liking and taste scores, suggesting that excessive protein cross‐linking may have produced an overly firm or less desirable texture. This finding indicates that instrumental texture enhancement does not necessarily correspond directly with consumer preference, highlighting the importance of optimizing TGase concentration to achieve a balance between technological functionality and sensory quality.

Similar findings have been reported in previous studies. Tseng [48] observed that low‐salt chicken meat balls containing TGase showed improved texture, juiciness, and overall liking compared with the control, although appearance, color, and flavor scores were unaffected. Dimitrakopoulou [32] also reported that TGase significantly improved the consistency and overall acceptability of restructured cooked pork shoulder, with the formulation containing 0.15% TGase receiving the highest consumer rating. In addition, TGase has been successfully applied in combination with other functional ingredients in healthier meat products. Zhang et al. [34] demonstrated increased consumer acceptance of reduced‐salt frankfurters containing TGase and lysine, while a salt‐free and low‐fat Dӧner kebab formulated with TGase and sodium caseinate received higher overall acceptability scores than the control product [59].

Overall, the present findings suggest that TGase can effectively improve the sensory quality and consumer acceptance of reduced‐fat and reduced‐sodium pork balls when applied at an appropriate concentration, with 0.1% TGase appearing to be the optimal level for balancing texture enhancement and sensory preference.

### 3.2. A Validation Study—Quality Analysis of the Reduced‐Fat and Reduced‐Sodium Pork Ball Containing 0.1% TGase Enzyme

Based on the consumer acceptability results (Table 4), the pork ball formulation containing 0.1% TGase was selected for further validation because it achieved the highest overall sensory scores, particularly for texture and overall liking. The selected product exhibited a pH value of 6.11 and contained 73.43 g/100 g moisture, 18.24 g/100 g protein, 6.46 g/100 g fat, and 2.05 g/100 g ash, whereas carbohydrate was not detected. The sodium content of the product was 337.7 mg/100 g. In terms of TPA, the product showed hardness, springiness, cohesiveness, and chewiness values of 12,661 g force, 0.73, 0.52, and 6,552 N.mm, respectively (Table 5), indicating desirable textural characteristics for emulsion‐type pork balls.

**Table 5:** Textural parameters and consumer acceptance of the reduced‐fat and reduced‐sodium pork ball containing 0.1% transglutaminase enzyme: a validation study.

**Textural parameters** ^**a**^	**Result**
Hardness (g force)	12,661 ± 1222
Springiness	0.73 ± 0.03
Cohesiveness	0.52 ± 0.02
Chewiness (N.mm)	6552 ± 572
**Consumer acceptance** ^**b**^	**Result**
Overall liking	6.9 ± 1.0
Salty	6.5 ± 1.1
Overall taste	6.8 ± 1.1
Texture	6.9 ± 1.2

^a^ ^a^Mean ± standard deviation based on 10 replications.

^b^ ^b^Mean ± standard deviation based on 200 consumer responses and on a 9‐point hedonic scale (1 = *dislike extremely*, 5 = *neither dislike nor like*, and 9 = *like extremely*; [40]).

Microbiological analysis demonstrated that the validated product was microbiologically safe for consumption. Pathogenic microorganisms including *Salmonella* spp., *S. aureus*, and *C. perfringens* were not detected, whereas *E. coli* levels were lower than 3 MPN/g. All microbial counts complied with the acceptable limits established by the Thai Industrial Standards Institute (TISI), Ministry of Industry [60]. In addition, the product received consumer acceptability scores ranging from 6.5 to 6.9, indicating that the product was generally liked by consumers.

Compared with conventional pork ball products, the optimized formulation containing 0.1% TGase achieved substantial reductions in both fat and sodium contents, with 47.5% less fat and 27.1% less sodium. According to the nutrient content claim regulations established by the United States Food and Drug Administration (FDA) [61], products may be labeled as “reduced fat” or “reduced sodium” when they contain at least 25% lower amounts of fat or sodium than an appropriate reference product. Therefore, the developed pork ball formulation satisfied the criteria for reduced‐fat and reduced‐sodium labeling claims.

These findings demonstrate that TGase incorporation at an appropriate level can successfully improve the quality and acceptability of healthier pork ball products while maintaining microbial safety and meeting nutritional labeling requirements.

## 4. Conclusion

This study demonstrated that TGase can be used to improve the textural and sensory quality of reduced‐fat and reduced‐sodium pork balls. Increasing TGase concentration generally enhanced hardness, springiness, cohesiveness, and chewiness, which can be attributed to TGase‐mediated protein cross‐linking and the development of a stronger protein network. Among the concentrations evaluated, 0.1% TGase (w/w of pork leg meat) was selected as the most suitable formulation because it provided improved textural characteristics and the highest consumer acceptability, particularly for texture and overall liking. These findings indicate that an appropriate level of TGase can help address some of the textural limitations associated with simultaneous reduction of fat and sodium in pork balls.

The selected 0.1% TGase formulation showed acceptable physicochemical and textural properties and met the microbiological safety criteria evaluated in this study. It also achieved reductions in fat and sodium of 47.5% and 27.1%, respectively, compared with the reference product, supporting its classification under the applicable United States FDA criteria for reduced‐fat and reduced‐sodium nutrient content claims. Overall, TGase has potential as a functional ingredient for developing reduced‐fat and reduced‐sodium pork balls with improved textural quality and consumer acceptability.

## Author Contributions

Jarinya Sutti: conceptualization, methodology, formal analysis, and writing—original draft. Sujinda Sriwattana: conceptualization, funding acquisition, methodology, formal analysis, project administration, resources, supervision, validation, visualization, writing—review and editing. Napapan Chokumnoyporn: methodology, formal analysis, validation, writing—original draft. Witoon Prinyawiwatkul: conceptualization, investigation, validation, writing—review and editing.

## Funding

This study was supported by the National Research Council of Thailand, 10.13039/501100004704; and the Graduate School, Chiang Mai University, 10.13039/501100007859.

## Ethics Statement

This research was a collaborative effort between Chiang Mai University and the Louisiana State University (LSU) Agricultural Center. The use of human subjects in research was approved by the Institutional Review Board, LSU Agricultural Center (IRBAG# 21‐0063; approved on October 1, 2024).

## Conflicts of Interest

The authors declare no conflicts of interest.

## Data Availability

The data generated during the current study are available from the corresponding author on reasonable request.
